# Love, jealousy, satisfaction and violence in young couples: A network analysis

**DOI:** 10.1371/journal.pone.0285555

**Published:** 2023-05-05

**Authors:** José Ventura-León, Cristopher Lino-Cruz

**Affiliations:** 1 Facultad de Ciencias de la Salud, Universidad Privada del Norte, Lima, Perú; 2 Facultad de Psicología, Universidad Peruana de Ciencias Aplicadas, Lima, Perú; Universidad Complutense de Madrid Facultad de Psicologia, SPAIN

## Abstract

In recent years, couples have been affected by health measures related to COVID-19, a circumstance that forces us to examine couple interactions in terms of crucial variables of their functioning. In this sense, the present study aimed to examine the association between love, jealousy, satisfaction, and violence in young couples through network analysis. A total of 834 young people and adults between 18 and 38 years of age (Mean = 20.97, SD = 2.39) participated; 646 women (77.50%) and 188 men (22.50%), who completed the Sternberg’s love scale (STLS-R), Brief Jealousy Scale (BJS), Relationship Assessment Scale (RAS) and Woman Abuse Screening Tool (WAST-2). A partial unregularized network was estimated using the ggmModSelect function. The Bridge Strength index was calculated because the aim was to identify the bridge nodes between the variables under study. The results reveal that two nodes of the love variable, Commitment, and Intimacy, had a direct and moderate relationship with the Satisfaction node. The latter is the central node in the network. However, in the male group, the most intense associations are in Satisfaction-Intimacy, Violence-Passion, Jealousy-Commitment. It is concluded that there are relevant connections between the nodes of the network, which invite further research on couple relationships after the COVID-19 pandemic.

## Introduction

In the last decades, an increase in the separation and divorce rates has been reported [1, 2]. In fact, in countries such as the United States and Canada a divorce culture is being discussed for some time already [3–5]. This might also be related to the decrease in the marriage rates registered in countries such as Ireland, Spain, Italy and Portugal in 2020 [6]. Consequently, Peru is not an exception. According to data from the National Institute of Statistics and Informatics [7], the separated and divorced population has been increasing exponentially, while married population has decreased in the period between the censuses of 1993 and 2017. In spite of the reported levels of divorce, young people are still in search of love relationships due to the fact that close relationships are the main contributing factor for personal happiness [8]. Thus, in the field of love relationships, literature has favored the study of love and satisfaction. However, emotions such as jealousy and rage have been studied recently [9].

It is known that the COVID-19 pandemic has had a negative impact in different aspects of people’s life, specifically in love relationships [10, 11]. In that sense, a decrease in satisfaction of young couples who do not live together was observed due to the little time shared with the partner [12]. Following that, the low satisfaction level in relationships has caused most marriages to end in divorce [13]. Thus, satisfaction in couples ends up being a key construct because it allows to assess the success and durability of the relationship [14]. Another important factor that helps to develop and maintain love relationships is love [15]. The construct is composed of three interrelated components: intimacy, passion, and commitment [16]. Therefore, love becomes a key element in love relationships, as it might hamper the self-realization of the individuals if it is not satisfied [17]. Regarding that, previous research prove a direct relation between love and satisfaction [18]. Moreover, some authors reported that satisfaction and love, both in dating and married couples, were considerably lower after the end of the COVID-19 emergency [19].

Another usual element in the field of love relationships is jealousy, because it is essentially a normal reaction in people [20]. Nevertheless, this construct takes on a varied emotional force because it can turn love into anger and tenderness into acts of control [21]. In fact, in some situations it can assume maladaptive dimensions when it is shown excessively. This harms the harmony of the relationship or even damages the physical and mental integrity through violence [20]. Regarding the forementioned, violence in love relationships refers to the attempt to dominate and control the other party, either physically, psychologically or sexually [22]. Although the research on violence in love relationships has focused on adult couples, it has been known for a while that it has strong implications in young couples [23]. The forementioned demands to keep exploring the love, satisfaction, jealousy, and violence variables in young couples, as no studies that relate these four variables have been found in literature.

Scientific evidence shows that the correlational studies with love and satisfaction [18, 19], jealousy and satisfaction [24], and aggression and jealousy variables [25] were performed using the Pearson correlation. However, no correlation studies with the network analysis were found, as it is known that this approach is considered as a novel and potentially more efficient method for the study of psychological attributes, unlike the model of latent variables [26, 27]. This is because its utility is greater for the study of the interaction between variables because it allows the researcher to examine the reciprocal association between different variables that belong to the same construct, as well as to assess the mutual interaction of different constructs [28]. Regarding the forementioned, with the network analysis a psychological attribute is a system in which all components interact with each other without being linked to a latent common cause [26]. In order to understand the network analysis approach, it is necessary to differentiate the following concepts: (a) *network*, refers to an abstract model that contains nodes and edges; (b) *nodes*, refer to the subjects or variables of the study; and (c) *edges*, refer to the connections between the nodes [29]. In this sense, nodes can have a strong relation witch each other, can be related to other facets within the network and/or act as bridges between them [30].

It is true that network analysis was first used in Psychology to explain psychopathological concepts [27], and, later, to analyze aspects of personality [31], intelligence [32], or even conspiracy beliefs [30]. Nevertheless, to date there is no evidence in literature to assess variables in the field of love relationships, specifically in young couples. In that sense, a void of knowledge related to the study of the variables of love, satisfaction, jealousy, and violence within the network analysis approach can be observed, especially in the Latin American context. This demands to use novel and effective methods, such as the network analysis, to analyze the study variables.

Therefore, the objective of the current research was to examine the structure of the network between the variables of love, satisfaction, jealousy, and violence in Peruvian young couples. Particularly, it aims to: (a) identify the interconnections between every node of the network; (b) recognize the main nodes; and (c) identify those nodes that function as bridges between other nodes.

## Materials and methods

### Participants

The participants were 834 young people and adults aged between 18 and 38 years (Mean = 20.97, *SD* = 2.39); 646 females (77.50%) and 188 males (22.50%). Every participant was in a love relationship of a minimum of three months, as this was considered as a necessary time for a certain stability [33]. Overall, the couple relationship time ranged between 3 and 139 months (Mean = 22.68, *SD* = 20.10). The size of the sample was estimated *a priori* through the *powerly* package setting 10 nodes, statistical power of 0.80, density of 0.40, which indicated a recommended minimum size of 276 observations [34]. The selection of the participants was carried out through nonprobability snowball sampling [35], because in Peru the massive and in-person applications have been affected after the COVID-19 pandemic.

#### Instruments

*Sternberg’s love scale* (STLS-R) was used in this study [36]. The Peruvian version of the STLS-R [37] is composed of fifteen items with a Likert-type scale that ranges from 1 (*Never*) to 5 (*Always*). It is a three-factor measure that explores intimacy, passion, and commitment. The validity obtained through confirmatory factor analysis obtained acceptable goodness of fit (CFI = .99; RMSEA = .03; SRMR = .03). The reliability coefficients estimated using omega, for the three factors: intimacy (ω = .91), passion (ω = .86), and commitment (ω = .93) can be considered good.

*Brief Jealousy Scale* (BJS) was used in this study [38]. EBC was a dimension of the Inventory of Emotional Communication in Love Relationships [39]. It is composed of nine items that are rated on a Likert-type scale from 1 (*Not at all jealous*) to 5 (*Very jealous*), and it explains a few scenarios in which someone might experience jealousy. Validity was obtained through confirmatory factor analysis, which revealed acceptable goodness of fit (CFI = .97; SRMR = .03; RMSEA = .08). Reliability was obtained through coefficient omega (ω = .88).

*Relationship Assessment Scale* (RAS) was used in this study [40]. The Peruvian version of the RAS uses a Likert-type response scale that ranges from 1 to 5 [41]. It is a one-dimensional instrument that assesses relationship satisfaction. Item response theory (IRT) and confirmatory factor analysis (CFA) were combined to determine the scale’s validity. In both approaches, it showed excellent goodness of fit (RMSEA < .08; CFI > .95). However, reliability was measured using two coefficients: empirical reliability (*r*_*xx*_ = .86) and coefficient omega (ω = .84), which indicated a good internal consistency.

*Woman Abuse Screening Tool* (WAST-2) was used in this study [42]. The Spanish version of the WAST-2 has two Likert-type format items make up this instrument [43]. It is a one-dimensional scale that assesses the presence of existing violent spells of tension and difficulty in the couple relationship. The validity was examined through validity evidence in relation to another variable; specifically, by contrasting scores of battered vs non-battered women, the results showed high sensitivity (91.4%) and specificity (76.2%). The estimated reliability for the sampling in the study exceeded 0.60 (ω = .66), a value that can be deemed acceptable for various studies [44–46]. This value signifies that more than 60% of the variance in the items can be attributed to their commonalities [47].

#### Procedures

Before conducting the study, the ethical considerations outlined in the Declaration of Helsinki [48] and aspects of doing online research [49] were evaluated. These ideas were given to the Research Ethics Committee of the Universidad Privada del Norte (UPN) in Peru. All participants were required to sign a consent form that outlined the aims of the study, the anonymity guarantee, the potential benefits and dangers, and the data treatment. Along with the questionnaires on couple relationships, a sociodemographic card was used. The average response time to the form was 12 minutes. Details of the R code can be found in S1 Appendix.

### Data analysis

In the RStudio environment [50], data analysis was conducted using the R programming language [51]. The present methodology was conducted by sequentially following a series of stages, which encompassed network estimation, precision analysis, stability evaluation, and comparative examination [52].

Before starting the network analysis, an exploratory analysis of the variables or nodes of interest was carried out. Descriptive statistical measures such as mean, standard deviation, minimum, maximum, skewness, kurtosis, and performance percentage were used to obtain detailed information about the fundamental characteristics of the variables.

In addition, as part of the network description, calculations of Global Network Properties were performed, including (a) density (D), which represents the proportion of connections existing in the graph; (b) transitivity (C^Δ^), which measures the average tendency of nodes to form groups or communities in the network; and (c) average shortest path length (APL), which indicates the average number of links or connections needed to reach from one node to another in the network. Finally, the small-world index (S) was calculated because some network structures may exhibit high node clustering but low APL. S evaluates the degree of association between nodes, with a value greater than 1 being recommended [53].

Network estimation was created with the function *ggmModSelect* and Spearman correlation. This is because it is assumed that data are asymmetric and, in those conditions, this combination produces better estimations [54]. In this point, the centrality indexes were examined. Due to the intention to examine nodes from different communities, the *Bridge Strength* (BS) index was preferred, which is the summatory of the *edges* (excluding the signs) that exist between a node and other nodes that are not in the same community [55]. Intermediation and closeness indices were not used because in some psychological networks such as romantic networks, the assumption that each node tries to connect with all other nodes in the network is not fulfilled. Moreover, the presence of negative edges makes interpretation difficult, since these indices were developed with the idea of distances and distances cannot be negative [56]. On the other hand, a simulation study found that Strength is a better indicator of an underlying latent variable and that closeness and betweenness are strongly affected by bridging nodes [57].

For the interpretation of the network, it should be considered that each node (circle) is connected to other nodes by edges (lines), whose thickness shows the strength of the interaction. The colors in green and red tones show positive and negative correlations respectively [58]. The organization of the nodes was made through the Fruchterman-Reingold algorithm, which places the stronger interactions at the center and the weaker ones at the periphery [59]. Predictability indexes are included to the estimations through R2. They indicate the percentage of explained variance of each node with the other nodes of the network [24].

The precision of the weights of the edges was estimated with the statistics resampling technique called bootstrapping through the *bootnet* package. This technique consists in repeatedly estimating a model with data randomly extracted from a data set and estimating a value for the edges in each attempt. This procedure allows to estimate confidence intervals (CI) to 95%, whose width indicates the precision of the edges [60]. A graph depicting the frequency with which the parameter is set to zero is also displayed.

Stability was examined through a graph that informs the variations in centrality indexes after removing 70% of the data and making a comparison between resampled data and the study data through the average of their correlations. A coefficient summarizes this process (CS, correlation of stability) that indicates the amount of data that can be removed to keep a correlation of at least 0.70 with the centrality coefficients of the data. The final value of the CS is expected to be between 0.25 ≤ CS ≥ 0.50 [58].

There was a comparison made by sex through the *NetworkComparisonTest* package [61]. NCT works under a procedure of permutations and examines the differences after one thousand randomly obtained replications assuming a null hypothesis that both groups are identical. Nevertheless, to assess the effect size, bootstrap-based Spearman correlations are established, and the average of the correlations obtained from one thousand resamples is reported. On addition, the differences are investigated through a subtraction of the values of both matrices, which are shown in a corPlot graph, allowing for the instant identification of the most significant differences between the two networks.

## Results

### Preliminary analysis of the variables

Table 1 shows the preliminary analyses of the variables. It is observed that, among the love dimensions, Intimacy shows the highest arithmetic mean and Passion shows the lowest. According to the performance percentage, Satisfaction and Jealousy are the most intense variables in the population, while the least intense is Violence. The minimum and maximum values are located within the expected ranges. Mostly, asymmetry is negative, except for Violence. This indicates an inclination toward higher scores. In terms of kurtosis, Intimacy is the variable with the largest data peaking.

**Table 1:** Descriptions of the study variables.

Variables	Mean	SD	Min.	Max.	g1	g2	%
Satisfaction	19.83	3.33	5	25	-0.76	1.00	79.34
Violence	1.54	1.02	0	4	0.19	-0.22	38.40
Jealousy	33.30	7.81	9	45	-0.82	0.38	74.01
Intimacy	25.33	3.79	6	30	-1.14	1.56	84.44
Passion	19.38	4.01	5	25	-0.66	0.06	77.52
Commitment	20.65	4.07	5	25	-0.96	0.44	82.59

*Note*. *SD*: Standard Deviation, g1: Skewness, g2: Kurtosis, %: Performance percentage.

### Global network properties

In terms of density, 9 out of 15 edges were non-zero, resulting in a density of 60 percent. The value of 0.47 was observed for transitivity, indicating a low proportion of closed triangles in the studied network. When compared to a random network, the observed transitivity is slightly higher (transitivity_random = 0.50). On average, about 1.4 links are required to reach from one node to another in the network, as indicated by APL, which is lower than the random APL (APL_random = 1.61). This suggests that the original network structure is more efficient in terms of communication. Finally, the small-world index obtained a value of 1.09, indicating that nodes are relatively close to each other, and information propagates efficiently in the network.

### Estimation of the network and centrality

**Fig 1:**
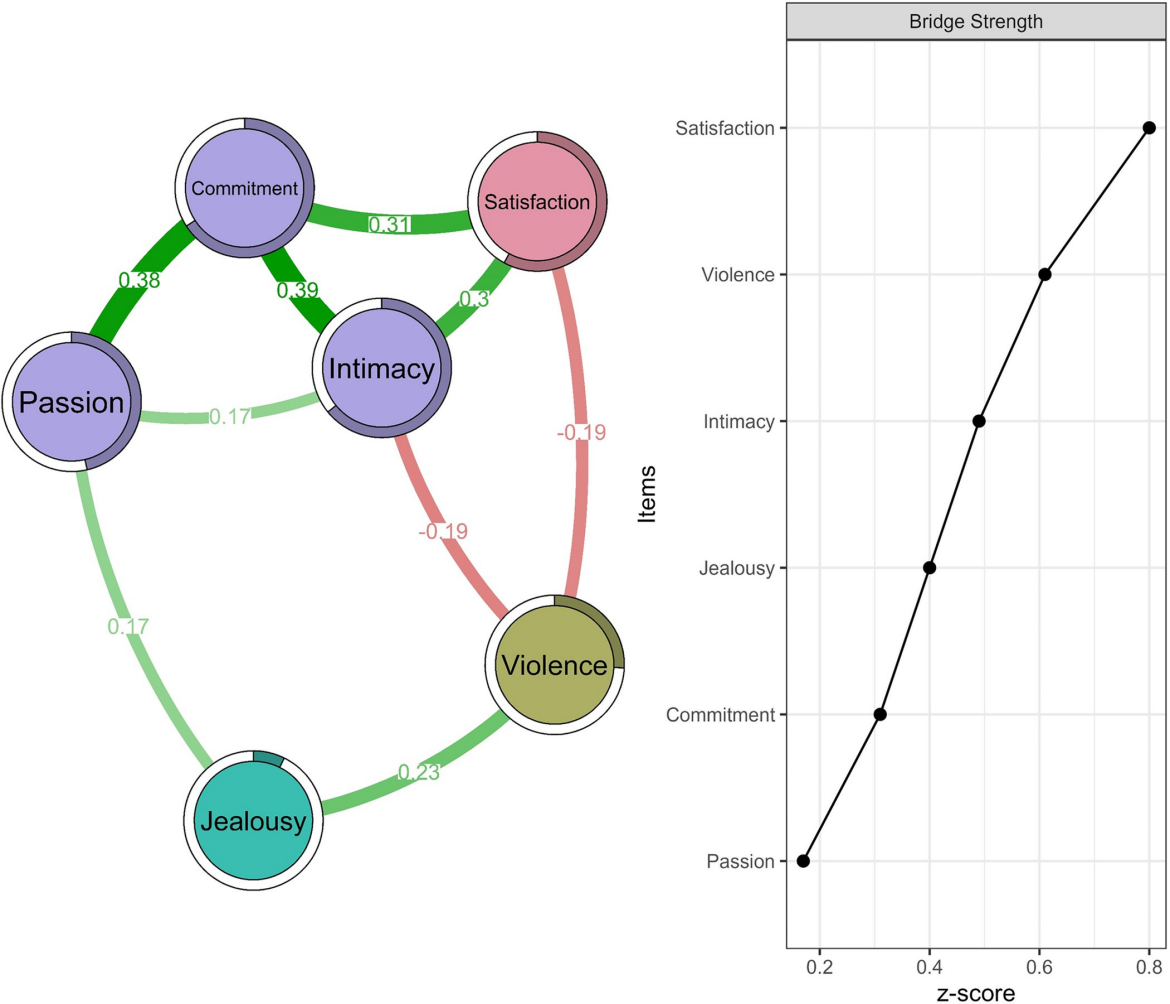


In Fig 1, the dimensions of the love variables are closer together, indicating the existence of a community or facet. It was discovered that the edges lie between -0.19 and 0.39. The Commitment-Satisfaction (*r* = .31) and Intimacy-Satisfaction (*r* = .30) edges showed a moderate and direct relation. That means, two of the three love factors are related to relationship satisfaction. On the other side, it is observed that violent events are similarly and inversely associated to Intimacy and Satisfaction (*r* = -0.19). In the case of Jealousy, a direct relation with Passion (*r* = .17) and Violence (*r* = .23) can be seen. These relations are consistent with the predictability measures obtained through R2 and that are visualized with a bar at the border of the circumference of the node.

### Stability and precision of the network

**Fig 2:**
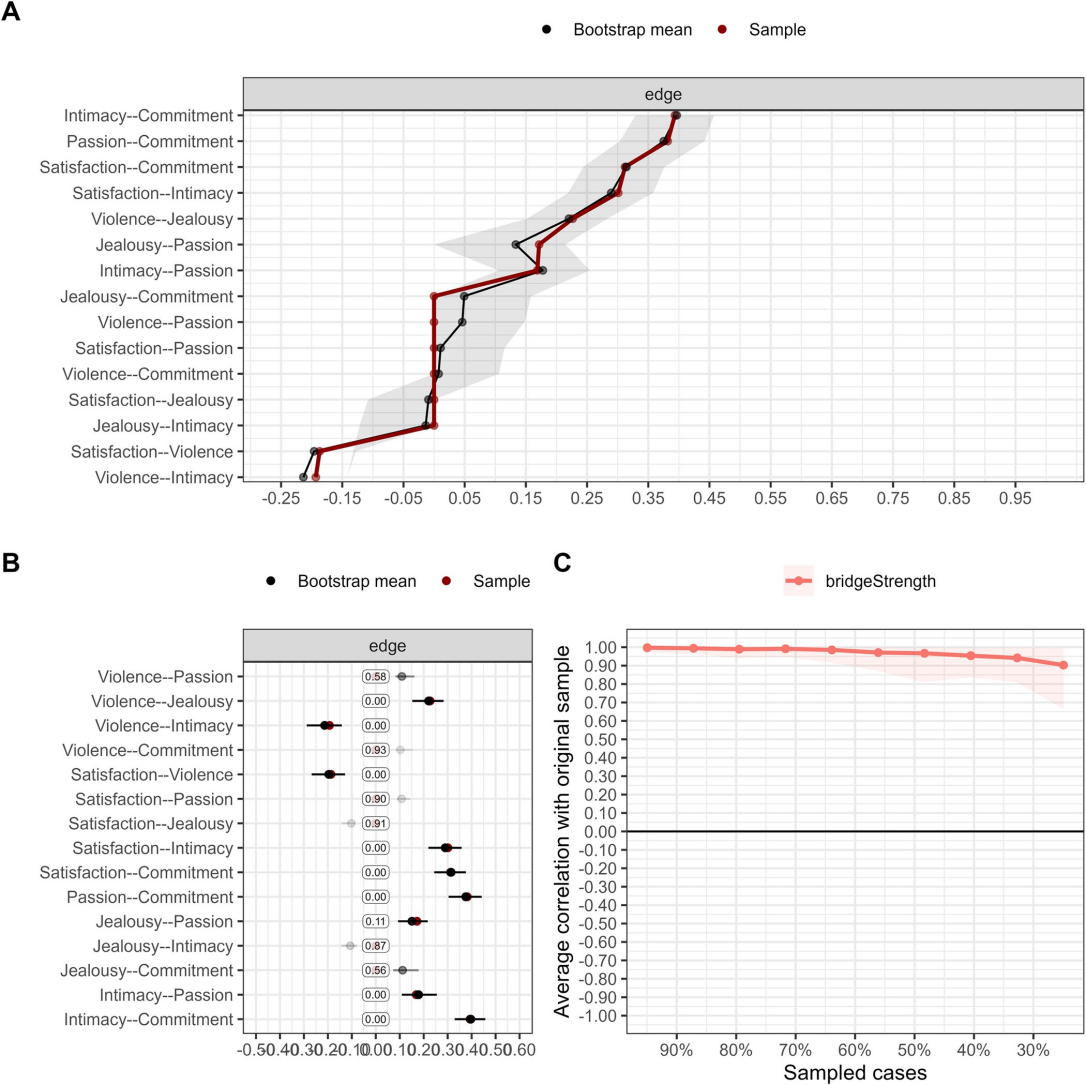


Fig 2A shows the precision of the edges through the comparison between the mean of the relation obtained in the resamplings (Bootstrap mean) and the ones obtained by the sample. An overlapping of the data can be seen (overlapped red and black line). In addition, the grey-color bar indicates that the confidence interval has little width, which suggests low variation between resamplings.

Fig 2B shows a graph that allows to see the times the parameter was not set in zero and its frequency. Consequently, Violence-Commitment was deleted from the network 92% of the time. Also, the transparency of the interval indicates that it was almost never included, but when it was, it was estimated in a small and positive magnitude. On the other hand, it is observed that Satisfaction-Commitment was never removed from the network. In addition, the black tone of the interval indicates that it was always included in the network and, when this happened, its estimation was close to 0.25.

Fig 2C shows the visualization of stability of the centrality index obtained through resampling. It is observed that the average correlation between the resampled and the original data is above 0.80 and this does not decrease even if cases are removed. Likewise, the coefficient of stability (CS) was 0.75, above the recommended minimum (CS > .50).

### Comparison

**Fig 3:**
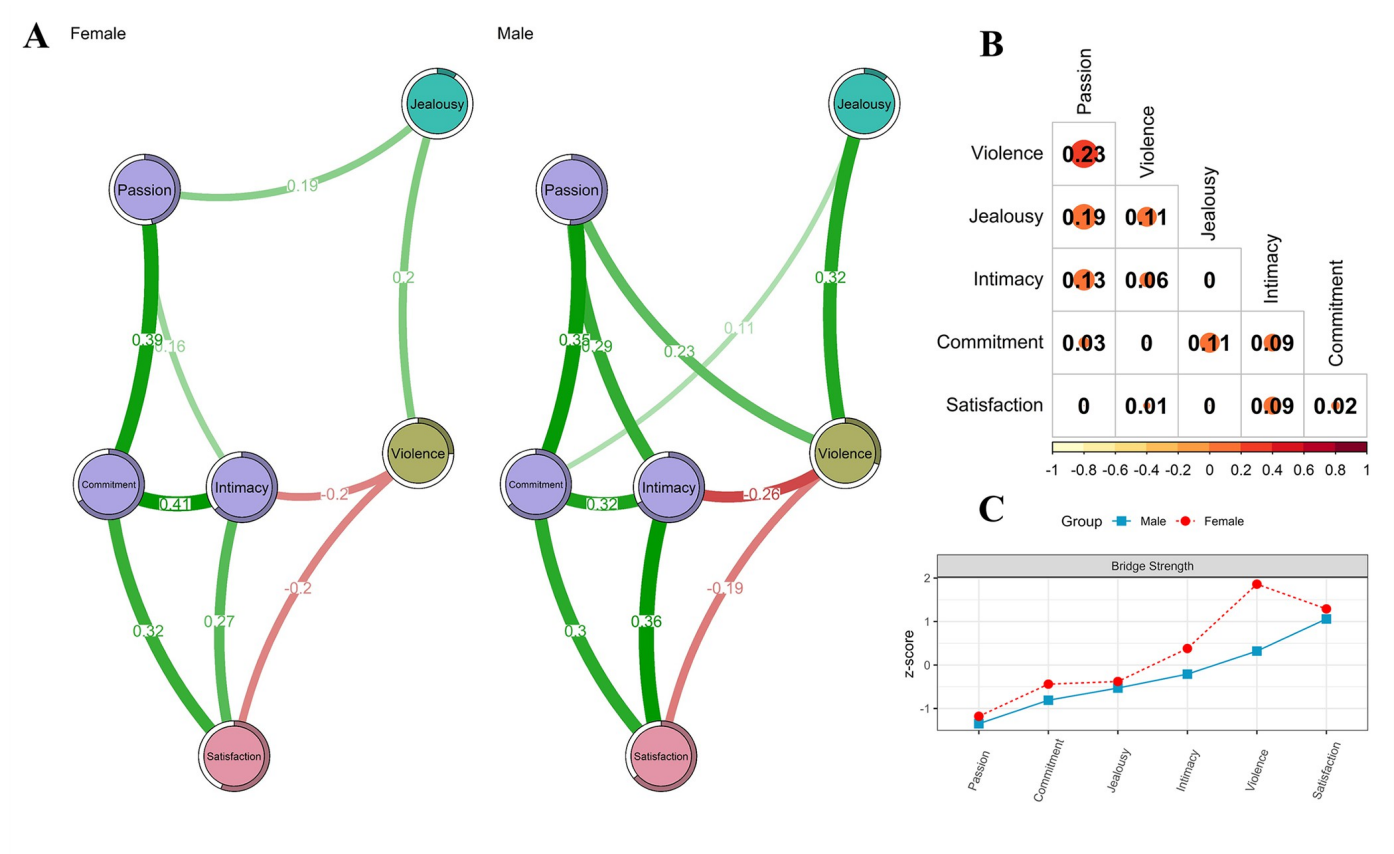


Fig 3 shows the comparisons of the networks by sex. In terms of statistical significance, the network seems invariant (*M* = 0.23; *p* = .310) and the connectivity is identical (*S* = 0.40; *p* = .147). Regarding the similarity of the edges, they are almost identical in the adjacent matrices. This is because, after generating 1000 matrices based on bootstrap for each network and obtaining their correlation in each of them, it was observed that in average the correlation was above 0.80 (*r*_s_ = .86). Additionally, the corPlot graph facilitated the swift identification of crucial differences in the interactions among constructs. Consequently, the males reveals a more robust correlation between Satisfaction and Intimacy (*r* = .36) compared to the females (*r* = .27). Furthermore, only males display a positive correlation between Violence and Passion (*r* = .23), in contrast to the females, where such correlation is absent. Finally, the males population exhibits a positive correlation between Jealousy and Commitment (*r* = .11), while in the females, this relationship is not found.

## Discussion

The current study is the first one to examine the relations between love, satisfaction, jealousy, and violence in young Peruvian couples with a network analysis approach. This approach was used because it allows to analyze the association between different nodes that belong to the same facet, as well as the relation between different facets [28, 58, 62]. Considering the lack of research in the field of love relationships with the network analysis approach, the interaction between the different variables of love relationships is examined. In this regard, Global Network Properties reported that the network has a more efficient structure than a random network with the same density, which translates into a more organized and cohesive network that facilitates the propagation of information between nodes.

The first objective was to identify the interconnections between each of the nodes of the network. It could be seen that two nodes of the love variable, Commitment, and Intimacy, had a direct and moderate relation with the Satisfaction node. In fact, this was expected, as commitment refers to the will of maintaining love over time and the decision of loving and being loved [16], and intimacy requires a degree of trust, closeness and connection with the partner [37, 63]. Hence, both concepts are key elements to maintain more satisfying relationships. In other words, love is related to satisfaction in love relationships, as mentioned in previous research [18, 19]. Thus, the forementioned is complemented with the idea that love and satisfaction assess the success and durability of love relationships [14, 15]. On the other hand, the nodes of Violence with Intimacy and Satisfaction show an inverse and moderate relation. These findings were expected if we consider that domestic violence refers to all forms of physical, psychological, sexual and economic abuse that occurs in the relationship [64]. Therefore, with the presence of any type of violence, it would be less probable for relationships to have confidence and closeness degrees or satisfying relationships. In that sense, any form of violence hinders the full development of the couple, basically because it reflects coercive methods and prevents to balance self and couple’s needs [65].

The second objective was to identify the central node that acts as a bridge in the network. Thus, the Satisfaction node was the most central in the love relationship community. These results indicate that satisfactions is a key element in the field of love relationships [14]. Although this study is not the first to establish the relation between love and satisfaction before [18] and during the pandemic [19], it is indeed the first to examine these interactions with the network approach. Another important finding is that Satisfaction has a direct relation with Passion and Violence, variables that could physically and mentally damage the love relationship [20]. Hence, it is necessary to keep exploring the love, satisfaction, jealousy, and violence variables in young couples, as no studies that relate these four variables have been found in literature.

A third objective was to compare the nodes by sex. The most evident implication from the analysis is that, globally, the networks are invariant, and the adjacent matrices are similar. However, research results indicate that men exhibit a stronger correlation between satisfaction and intimacy in relationships compared to women. This difference may be due to various causes, including gender differences in experiencing and expressing emotions, as evidenced in previous studies [66]. Specifically, men tend to report feelings of calmness and excitement more frequently, which may be subject to cultural expectations about masculinity and success. Additionally, men exhibit a positive correlation between violence and passion, unlike women, suggesting that men may show greater expressions of dominance and control over their partners, which has often been identified as a trigger for violence against women [67, 68]. These results highlight the importance of addressing the problem of partner violence from a gender perspective. Finally, men show a positive correlation between jealousy and commitment, whereas no such relationship is found in women. While a small amount of jealousy can be an indicator of love in a relationship, it can also represent a threat to the relationship when it intensifies and leads to conflicts and mistrust [20, 69]. It should be noted that the presence of jealousy in romantic relationships appears to be more common in men than in women, as observed in previous studies [70]. These results contribute to a better understanding of gender factors that influence emotions and behaviors in relationships.

The results of this investigation have strong theoretical and practical implications. In fact, the fact that centrality is in Satisfaction supports the models that highlight the role of this construct, which is involved in the success and durability of the relationship [14]. These results show that the COVID-19 pandemic has had a negative impact in love relationships [10, 11] due to the little time shared in couples that do not live together [12]. Also, in the case of marriages, it has led to their culmination [13]. In a practical way, the results highlight the importance of considering couple satisfaction within the prevention plans that, along with Commitment and Intimacy, can serve as protecting factors against divorce or separation. This is important because the pandemic modified the interpersonal relationships with the distancing measures. Finally, it is important to note that jealousy interacts with the passional element of love and violence. Also, due to love relationships being composed of control and dominance in the present society [22], it is necessary to address both positive and negative interactions in order to contribute to the understanding of love relationships.

The results of this research are subject to the following limitations. Firstly, the participants were obtained with a snowball-type non-probability design because it was the only way of collection during the time of the research. In fact, there is evidence to assume that in Psychology non-probability designs are the most frequently used [71]. Moreover, the application of a random sampling is complex in a virtual context. Secondly, the sample inequality between men and women are a result of the sampling design, as there is no control of the proportionality of the sample. In spite of that, it is recommended for future studies to pursue the equivalence between men and women for the comparison by networks.

## Conclusion

In conclusion, the objective of the articles was to examine the interaction between the elements of love, relationship satisfaction, jealousy and violence in a sample of young Peruvians. The results indicate that the nodes of Commitment and Intimacy had a direct and moderate relation with the Satisfaction node, while Violence showed an inverse relation with Intimacy and Satisfaction. In second place, it was determined that Satisfaction is the core node that works as a bridge. This places this variable as one of the most influential in love relationships. Thirdly, when comparing by gender, it is observed that males exhibit larger correlations between Satisfaction-Intimacy, Violence-Passion, Jealousy-Commitment. However, these results should be taken with caution due to the disproportionality of the subsamples. These findings encourage the continuation of research on love relationships in order to better understand their dynamic, which has been affected after the COVID-19 pandemic in benefit of a healthier society.

## Supporting information

S1 Appendix R codes used in data analysis. (DOCX)
