# Supplementary material for: Love, jealousy, satisfaction and violence in young couples: A network analysis
Source: PLoS One. 2023 May 5;18(5):e0285555. doi: 10.1371/journal.pone.0285555 (PMC10162535; doi:10.1371/journal.pone.0285555)
Supplement: S1 Appendix — (DOCX) [file pone.0285555.s001.docx]

**Appendix S1: R codes used in data analysis**

Load the libraries to be used

library(readxl)

## Warning: package 'readxl' was built under R version 4.1.3

library(tidyverse)

library(bootnet)

library(qgraph)

library(ggplot2)
library(NetworkComparisonTest)

library(networktools)

library(igraph)

library(mgm)

library(psych)

Loading database

df <- readxl::read_excel("love_data.xlsx")

Description of participants

#Age
summarytools::descr(df$Age)

#Sex
df %>% count(Sex) %>%
 mutate(Porcentaje = n/sum(n)*100)

# Type of relationship
df %>%
 summarytools::descr(df$Time_relationship)

Determining the sample size (a priori)

library("powerly")

## Warning: package 'powerly' was built under R version 4.1.3

set.seed(1234)

density.net = list()
for (i in 1:10) {
set.seed(2022)
results <- powerly(
 range_lower = 300,
 range_upper = 1000,
 samples = 30,
 replications = 100,
 measure = "sen",
 statistic = "power",
 measure_value = .6, # sensitivity
 statistic_value = .8, # probability power
 model = "ggm",
 nodes = 10, # nodes
 density = .4, # density
 cores = 12,
 verbose = TRUE
)
true_model <- generate_model(type = "ggm", nodes = 10, density = .4) # density
results <- powerly(
 range_lower = 100,
 range_upper = 2000,
 samples = 30,
 replications = 100,
 measure = "sen",
 statistic = "power",
 measure_value = .6, # sensitivity
 statistic_value = .8, # probability power
 model = "ggm",
 cores = 12,
 model_matrix = true_model,
 verbose = TRUE
)
# Validate the recommendation obtained during the analysis.
validation <- validate(results, cores = 12)$recommendation
density.net[[i]] = validation
print(validation)
}

map_dfr(density.net, ~as_tibble(.)) %>%
 summarise(Media = mean(value))

## # A tibble: 1 x 1
## Media
## <dbl>
## 1 272.

Generate variables

df <- df %>%
 rowwise() %>%
 mutate(Satisfaction = sum(c_across(c(RAS1:RAS5))),
 Violence = sum(c_across(c(WAST1:WAST2))),
 Jealousy = sum(c_across(c(ECP1:ECP9))),
 Intimacy = sum(c_across(c(ETAS1,ETAS2,ETAS5,ETAS8,ETAS11,ETAS9))),
 Passion = sum(c_across(c(ETAS4,ETAS6,ETAS14,ETAS15,ETAS16))),
 Commitment = sum(c_across(c(ETAS3,ETAS7,ETAS10,ETAS12,ETAS13)))
 ) %>%
 ungroup()

Descriptive statistics

df %>% select(Satisfaction:Commitment) %>% psych::describe() %>%
 openxlsx::write.xlsx(., file = " Tabla 1.xlsx", overwrite = T, rowNames = T)

Organization of the communities

df_new <- df %>% select(Satisfaction:Commitment)
groups <- structure(list( `Satisfaction` = c(1),
 `Violence` = c(2),
 `Jealousy` = c(3),
 `Love` = c(4:6)
 ),
 Names = c("Satisfaction",
 "Violence",
 "Jealousy",
 "Love"))

Estimate the network with bootnet’s ggmModSelect.

# pak::pkg_install("r-lib/rlang")
network <-
estimateNetwork(df_new,
 default = 'ggmModSelect',
 stepwise = TRUE,
 corMethod = "spearman",
 tuning = 0,
 criterion = "ebic")

Generate R2 with mgm

type = c(rep("g", 6))
level= c(rep(1, 6))

mgm <- mgm(data = df %>% select(Satisfaction:Commitment),
 type = c(rep("g", 6)), ## "c"" indicates that the variable is categorical
 levels = c(rep(1, 6)), ## three indicates the number of levels of the variables
 k = 2)

## Note that the sign of parameter estimates is stored separately; see ?mgm

pred_Model <- predict(object = mgm,
 data = df %>% select(Satisfaction:Commitment),
 errorCon = c("RMSE", "R2"),
 errorCat = c("CC", "nCC"))

error_Model <- pred_Model$errors %>% select(Variable, R2) %>% pull(R2) %>% abs()
error_Model

## [1] 0.579 0.257 0.080 0.641 0.464 0.658

plot

#3. Elabore el gráfico de red en alta calidad y guarde en la pc
jpeg(""./Figuras Love/Figura 1 - Izquierda.jpg",
 width=6.5, height=8.5,
 units='in',res=1500)
g1 <- qgraph(network$graph,
 groups = groups,
 curveAll = 2,
 vsize = 18,
 esize = 18,
 palette = "pastel", #colorblind #ggplot2 #pastel #rainbow
 layout = "spring",
 edge.labels = T,
 legend.cex = 0.45,
 legend = F,
 details = F,
 node.width = 0.8,
 pie = error_Model,
 layoutScale =c(0.8,0.8),
 labels = colnames(df %>% select(Satisfaction:Commitment)),
 edge.label.cex = 1)
dev.off()

## png
## 2

centrality Bridge Expected Influence

library("networktools")
b <-bridge(g1, communities= groups, useCommunities = "all", normalize = F)
# cents <- as.data.frame(cbind(b$`Bridge Expected Influence (1-step)`,
# b$`Bridge Expected Influence (2-step)`,
# b$`Bridge Strength`))

cents <- as.data.frame(cbind(b$`Bridge Strength`))
cents <- cents %>%
 mutate(cents, id = rownames(cents)) %>%
 rename_at(vars(V1:id), ~ c("Bridge Strength", "Symptoms")) %>%
 reshape2::melt(., id = "Symptoms") %>%
 # mutate(zscore = -1*value) %>%
 rename(Centrality = variable) %>%
 mutate(across(where(is.numeric), round, 2))

cents$overall = "Bridge Strength"
Figura1_Derecha <-
ggplot(cents,
 aes(x = forcats::fct_reorder(factor(Symptoms), value, mean), y = value,
 linetype=Centrality, shape = Centrality, group=Centrality))+
 geom_line()+
 geom_point(size =2.5)+
 # geom_text(aes(label=sprintf("%0.2f", value)), position=position_dodge(width=0.9), vjust=-0.25, size=3)+
 xlab("Items") +
 ylab("z-score")+
 theme_bw()+
 coord_flip()+
 facet_wrap(~overall)+
 theme(legend.position = "none")
Figura1_Derecha

ggsave(filename = "./Figuras Love/Figura 1.1 Derecha.jpg", plot = Figura1_Derecha,
 height = 6.5, width = 3.5, dpi = 600, units = c("in"))

Correlation matrices

#Spearman
SPcor <- function(x, ...) cor(x, method = "spearman", ...)
cor.matrix <- round(SPcor(df_new), digits = 2)
cor.matrix

#get edge weights
edge.matrix <- round(getWmat(getWmat(network$graph)), digits = 2)
edge.matrix

#Descriptivos de la matriz

Density

# Total de conexiones
Densidad_Grafo <- function(edge.matrix){
 n <- nrow(edge.matrix)
 Total_Density <- n*(n-1)/2
 Conexiones_Diff_cero <- sum(getWmat(edge.matrix) != 0)/2
 Densidad <- Conexiones_Diff_cero/Total_Density*100
 resultado <- paste(Conexiones_Diff_cero, " of ", Total_Density, " edges were distinct from zero (", format(Densidad, digits = 2, nsmall = 2), "% of density).", sep = "")
 return(resultado)
}

Global metrics

smallworldIndex(qgraph(edge.matrix)) %>%
 map_df(., ~as_tibble(.), .id = "nombre_lista")

Estimate network stability

# 1. Bootstrap network estimation
caseDroppingBoot <- bootnet(network,
 boots=1000,
 type="case",
 nCores = 12,
 statistics="all",
 default='ggmModSelect',
 corMethod = "spearman",
 communities=groups)

data.frame(Index = round(CorStability,2)) %>%

rownames_to_column() %>%

filter(rowname == "bridgeExpectedInfluence"|
 rowname == "bridgeStrength"|
 rowname == "expectedInfluence"|
 rowname == "strength")

Estimate the accuracy of the network

# 1. Estimate the precision or accuracy of the network
nonParametricBoot <- bootnet(network,
 boots=1000,
 nCores = 12,
 default='ggmModSelect',
 corMethod = "spearman",
 stepwise = TRUE,
 type="nonparametric",
 statistics="all",
 communities=groups)

Combining Stability and Accuracy

# Plot centrality stability:
p1 <- plot(caseDroppingBoot, statistics = c("bridgeStrength")) +
 ggplot2::scale_y_continuous(limits = c(-1, 1), breaks = seq(-1, 1, by = 0.10),
 labels = scales::number_format(accuracy = 0.01))

## Scale for y is already present.
## Adding another scale for y, which will replace the existing scale.

p2 <- plot(nonParametricBoot, labels = T, order = 'sample',statistics = 'edge') +
ggplot2::scale_x_continuous(limits = c(-0.25, 1), breaks = seq(-0.25, 1, by = 0.10),
 labels = scales::number_format(accuracy = 0.01))

p3 <- plot(nonParametricBoot, plot = "interval", split0 = TRUE)+
 ggplot2::scale_x_continuous(limits = c(-0.50, 0.60), breaks = seq(-0.50, 0.60, by = 0.10),
 labels = scales::number_format(accuracy = 0.01))
p3

#library for joining plots
library("ggpubr")

## Warning: package 'ggpubr' was built under R version 4.1.3

#combinar los plots
p123 <- ggarrange(
 p2, # First row with line plot
 # Second row with box and dot plots
 ggarrange(p3,p1, ncol = 2, labels = c("B", "C")),
 nrow = 2,
 labels = "A" # Label of the line plot
 )
#guardar en alta calidad
ggsave(filename = "./Figuras Love/Figura 2.jpg", plot = p123,
 height = 8, width = 8, dpi = 600)

Comparisons by sex - R2 errors

Redes_Sexo = df %>%
 select(Sex, Satisfaction:Commitment) %>%
 group_nest(Sex) %>%
 mutate(
 data = map(data,
 ~ select(., tidyselect:::where(~ !all(is.na(.))))),
 Estimacion = map(data, ~ mgm(.x,
 type = c(rep("g", 6)),
 level = c(rep(1,6)),
 k = 2)))

error <- lapply(1:2, function(i) {
 pred <- predict(object = Redes_Sexo$Estimacion[[i]],
 data = Redes_Sexo$data[[i]],
 errorCon = c("RMSE", "R2"))
 error <- pred$errors %>% as_tibble() %>% select(Variable, R2) %>% pull()
 return(error)
})

Estimates

#generation of a specific data
Femenino= df %>%
 filter(Sex == "Mujer") %>%
 select(Satisfaction:Commitment) %>%
 na.omit() %>%
 as.data.frame()

Masculino = df %>%
 filter(Sex == "Varon") %>%
 select(Satisfaction:Commitment) %>%
 na.omit() %>%
 as.data.frame()

#Estimation of the subgroups (yes and no)
network_Female <- estimateNetwork(Femenino,
 default = 'ggmModSelect',
 stepwise = TRUE,
 corMethod = "spearman",
 tuning = 0
)

network_Male <- estimateNetwork(Masculino,
 default = 'ggmModSelect',
 stepwise = TRUE,
 corMethod = "spearman",
 tuning = 0
)

L <- averageLayout(network_Female,network_Male)

plot

#plot of both subgroups
jpeg("./Figuras Love/Figura3 Izquierda.jpg",
 width=8.5, height=8.5,
 units='in',res=1000)
layout(t(1:2))
g3 <- qgraph(network_Female$graph,
 layout = L,
 vsize = 10, #tama?o de los nodos
 palette = "pastel",
 groups = groups,
 legend = F, #Cambien aqu?
 edge.labels = T,
 edge.label.cex = 1,
 curveAll = 2,
 pie = error[[1]],
 cut = 0.00,
 labels = colnames(df %>% select(Satisfaction:Commitment)),
 title = "Female")
g4 <- qgraph(network_Male$graph,
 layout = L,
 vsize = 10, #tama?o de los nodos
 groups = groups,
 palette = "pastel",
 legend = F,
 edge.labels = T, #Cambien aqu?
 edge.label.cex = 1,
 cut = 0.00,
 labels = colnames(df %>% select(Satisfaction:Commitment)),
 curveAll = 2,
 pie = error[[2]],
 title = "Male")
dev.off()

Centrality index figure (Bridge Expected Influence)

c <- bridge(g3, communities= groups, useCommunities = "all", normalize = F)
d <- bridge(g4, communities= groups, useCommunities = "all", normalize = F)


cents_cd <- as.data.frame(cbind(c$`Bridge Strength`,
 d$`Bridge Strength`))

cents_cd <- cents_cd %>%
 mutate(cents_cd, id = rownames(cents_cd)) %>%
 rename_at(vars(V1:id), ~ c('Male','Female',"Symptoms")) %>%
 reshape2::melt(., id = "Symptoms") %>%
 mutate(zscore = scale(value)) %>%
 rename(Group = variable) %>%
 mutate(across(where(is.numeric), round, 2))
cents_cd

#Gráfico de puente ordenado
cents_cd$overall = "Bridge Strength"
Figure3_Derecha <-
ggplot(cents_cd, aes(x = forcats::fct_reorder(factor(Symptoms), zscore, mean), y = zscore, group=Group)) +
 geom_line(aes(linetype=Group, color=Group)) +
 geom_point(aes(shape = Group, color=Group), size = 3) +
 scale_shape_manual(values=c(15, 16)) +
 scale_colour_manual(values=c("#0C99C6", "red")) +
 xlab(" ") + ylab("z-score")+
 theme_bw() +
 theme(legend.position="top")+
 theme(axis.text.x = element_text(angle = 70, vjust = 0.5, hjust=0.5))+
 facet_wrap(~overall)

Figure3_Derecha

ggsave(filename = "./Figuras Love/Figure3 Derecha.jpg", plot = Figure3_Derecha,
 height = 8.81, width = 12.55, dpi = 600, units = c("cm"))

Comparisons using NCT

set.seed(2022)
library(NetworkComparisonTest)
res <-NCT(network_Female, network_Male,
 binary.data=F,
 test.edges=TRUE,
 edges="all",
 it = 1000,
 test.centrality = TRUE,
 verbose = FALSE)

#NETWORK INVARIANCE TEST
res$nwinv.real # Test statistic M:

res$nwinv.pval #p-value

#GLOBAL STRENGTH INVARIANCE TEST
res$glstrinv.real #Test statistic S

res$glstrinv.pval #p-value

Matrix effect size

#Calculation of effect size - Comparing adjacent matrices
network1_adjacency <- getWmat(network_Female)
network2_adjacency <- getWmat(network_Male)

cor(network1_adjacency[lower.tri(network1_adjacency)], network2_adjacency[lower.tri(network2_adjacency)], method = "spearman")

## [1] 0.8808385

#Calculation of effect size from bootstrap
compare_matrices_bootstrap <- function(network1_adjacency, network2_adjacency, num_repetitions) {
 library(boot)

 spearman_correlation <- function(mat1, mat2, indices) {
 mat1_vec <- as.vector(mat1[indices])
 mat2_vec <- as.vector(mat2[indices])
 return(cor(mat1_vec, mat2_vec, method = "spearman"))
 }

 set.seed(2023)

 lower_tri_indices <- lower.tri(network1_adjacency)

 network1_adjacency_vec <- as.vector(network1_adjacency[lower_tri_indices])
 network2_adjacency_vec <- as.vector(network2_adjacency[lower_tri_indices])

 # Almacenar las correlaciones de Spearman
 spearman_correlations <- numeric(num_repetitions)

 boot_obj <- boot(data = cbind(network1_adjacency_vec, network2_adjacency_vec),
 statistic = function(data, indices) {
 spearman_correlation(network1_adjacency_vec, network2_adjacency_vec, indices)
 },
 R = num_repetitions)

 mean_correlation <- mean(boot_obj$t)

 similarity_status <- if (mean_correlation > 0.8) {
 "Las matrices son similares."
 } else if (mean_correlation < 0.2) {
 "Las matrices son diferentes."
 } else {
 "Las matrices tienen alguna similitud pero también tienen diferencias."
 }

 return(list(
 spearman_correlations = boot_obj$t,
 mean_correlation = mean_correlation,
 similarity_status = similarity_status
 ))
}

# Uso de la función
result <- compare_matrices_bootstrap(network1_adjacency, network2_adjacency, 1000)

corrplot of differences between matrices

differences <- abs(getWmat(network_Female)-getWmat(network_Male))
library(corrplot)

## corrplot 0.92 loaded

jpeg("./Figuras Love/Figura4.jpg",
 width=10.8, height=10.8,
 units='cm',res=1000)
corrplot(differences, method = 'circle', type = 'lower', insig='blank', tl.col = 'black',
 addCoef.col ='black', number.cex = 1.2, order = 'FPC', diag=FALSE, col = COL1('YlOrRd', 10))
dev.off()

library information

sessionInfo()

## R version 4.1.2 (2021-11-01)
## Platform: x86_64-w64-mingw32/x64 (64-bit)
## Running under: Windows 10 x64 (build 22000)
##
## Matrix products: default
##
## locale:
## [1] LC_COLLATE=Spanish_Peru.1252 LC_CTYPE=Spanish_Peru.1252
## [3] LC_MONETARY=Spanish_Peru.1252 LC_NUMERIC=C
## [5] LC_TIME=Spanish_Peru.1252
##
## attached base packages:
## [1] stats graphics grDevices utils datasets methods base
##
## other attached packages:
## [1] corrplot_0.92 boot_1.3-28
## [3] ggpubr_0.6.0 powerly_1.8.6
## [5] psych_2.3.3 mgm_1.2-13
## [7] igraph_1.3.4 networktools_1.5.0
## [9] NetworkComparisonTest_2.2.1 qgraph_1.9.4
## [11] bootnet_1.5 lubridate_1.9.0
## [13] timechange_0.2.0 forcats_1.0.0
## [15] stringr_1.5.0 dplyr_1.0.10
## [17] purrr_1.0.1 readr_2.1.4
## [19] tidyr_1.3.0 tibble_3.1.8
## [21] ggplot2_3.4.2 tidyverse_2.0.0
## [23] readxl_1.4.1
##
## loaded via a namespace (and not attached):
## [1] snow_0.4-4 backports_1.4.1 Hmisc_4.7-2
## [4] systemfonts_1.0.4 plyr_1.8.8 splines_4.1.2
## [7] candisc_0.8-6 pryr_0.1.5 digest_0.6.29
## [10] foreach_1.5.2 htmltools_0.5.4 magick_2.7.3
## [13] gdata_2.18.0.1 fansi_1.0.3 magrittr_2.0.3
## [16] checkmate_2.1.0 cluster_2.1.2 doParallel_1.0.17
## [19] openxlsx_4.2.5.1 tzdb_0.3.0 wordcloud_2.6
## [22] matrixStats_0.63.0 R.utils_2.12.2 prettyunits_1.1.1
## [25] jpeg_0.1-10 colorspace_2.0-3 textshaping_0.3.6
## [28] xfun_0.36 crayon_1.5.2 tcltk_4.1.2
## [31] lme4_1.1-28 survival_3.2-13 iterators_1.0.14
## [34] glue_1.6.2 gtable_0.3.3 nnls_1.4
## [37] NetworkToolbox_1.4.2 car_3.1-2 weights_1.0.4
## [40] shape_1.4.6 abind_1.4-5 rapportools_1.1
## [43] scales_1.2.1 mvtnorm_1.1-3 DBI_1.1.3
## [46] rstatix_0.7.2 Rcpp_1.0.8.3 plotrix_3.8-2
## [49] progress_1.2.2 htmlTable_2.4.1 foreign_0.8-84
## [52] proxy_0.4-27 Formula_1.2-5 splines2_0.4.7
## [55] stats4_4.1.2 heplots_1.4-2 glmnet_4.1-6
## [58] htmlwidgets_1.6.2 RColorBrewer_1.1-3 lavaan_0.6-15
## [61] IsingFit_0.3.1 mice_3.15.0 farver_2.1.1
## [64] pkgconfig_2.0.3 R.methodsS3_1.8.2 nnet_7.3-18
## [67] deldir_1.0-6 utf8_1.2.2 labeling_0.4.2
## [70] tidyselect_1.2.0 rlang_1.1.0 reshape2_1.4.4
## [73] polynom_1.4-1 munsell_0.5.0 cellranger_1.1.0
## [76] tools_4.1.2 osqp_0.6.0.7 cli_3.6.0
## [79] generics_0.1.3 IsingSampler_0.2.1 broom_1.0.4
## [82] fdrtool_1.2.17 evaluate_0.20 summarytools_1.0.1
## [85] fastmap_1.1.0 ragg_1.2.5 yaml_2.3.6
## [88] knitr_1.42 zip_2.2.2 pander_0.6.5
## [91] glasso_1.11 pbapply_1.7-0 nlme_3.1-153
## [94] R.oo_1.25.0 smacof_2.1-5 compiler_4.1.2
## [97] rstudioapi_0.14 png_0.1-8 ggsignif_0.6.4
## [100] e1071_1.7-12 pbivnorm_0.6.0 stringi_1.7.6
## [103] highr_0.10 eigenmodel_1.11 lattice_0.20-45
## [106] Matrix_1.5-3 nloptr_2.0.0 vctrs_0.6.1.9000
## [109] pillar_1.9.0 lifecycle_1.0.3 cowplot_1.1.1
## [112] data.table_1.14.2 corpcor_1.6.10 patchwork_1.1.2
## [115] R6_2.5.1 latticeExtra_0.6-30 gridExtra_2.3
## [118] codetools_0.2-18 MASS_7.3-55 gtools_3.9.4
## [121] assertthat_0.2.1 withr_2.5.0 mnormt_2.1.0
## [124] parallel_4.1.2 hms_1.1.3 quadprog_1.5-8
## [127] grid_4.1.2 rpart_4.1.19 class_7.3-20
## [130] minqa_1.2.4 rmarkdown_2.21 carData_3.0-5
## [133] base64enc_0.1-3 ellipse_0.4.3 interp_1.1-3
